# Overexpression of EXO70E2 in *Arabidopsis thaliana* Disrupts Normal Development and Enhances Susceptibility to the Necrotrophic Fungus *Botrytis cinerea*

**DOI:** 10.3390/genes17030347

**Published:** 2026-03-20

**Authors:** Xiaoqiu Wu, Jianzhong Huang

**Affiliations:** 1 Puai Medical College, Shaoyang University, Shaoyang 422000, China; wuxiaoqiu@whu.edu.cn; 2 Department of Basic Medicine, Fuzhou Medical College, Fuzhou 344000, China; 3 College of Life Sciences, Wuhan University, Wuhan 430072, China

**Keywords:** *Arabidopsis thaliana*, EXO70E2, *Botrytis cinerea*, disease susceptibility

## Abstract

**Background:** The exocyst complex, a conserved hetero-octameric complex including the EXO70 subunit, is a pivotal regulator of various cellular and developmental processes in plants. Among the diverse EXO70 isoforms, the specific function of *EXO70E2*—a primary organizer of exocyst-positive organelles (EXPOs)—remains to be fully elucidated. **Methods:** Here, we investigated the functional impact of constitutive *EXO70E2* overexpression in *Arabidopsis thaliana*. **Results:** We observe that *EXO70E2* overexpression leads to severe etiolation and dwarfism, accompanied by dose-dependent inhibition of primary root elongation. The YFP-labeled EXO70E2 localizes to distinct punctate structures. Histochemical analysis shows *EXO70E2* expression in root tips and leaf vasculature, and its promoter activity is strongly induced by the salicylic acid analog INA and pathogen infection. Further function dissection demonstrates that *EXO70E2*-overexpressing plants exhibit enhanced susceptibility to the necrotrophic fungus *Botrytis cinerea*, manifested as larger lesions, accelerated host cell death, and increased fungal biomass. **Conclusions:** Our findings position EXO70E2 as a key negative regulator of plant development and defense outcomes, which may play a role in the balance between growth and immunity.

## 1. Introduction

In sessile plants, survival and ecological adaptability depend on the dynamic coordination of growth and development with adaptive responses to environmental stresses [1]. This coordination is controlled by sophisticated cellular logistics, and vesicle trafficking serves as a central regulatory mechanism [2]. Polarized exocytosis transports substances including cell wall components, membrane proteins, and signaling molecules to specific membrane domains, thereby supporting basic processes such as cell expansion, cytoplasmic division, establishment of cell polarity, and rapid pathogen response [3]. The exocyst complex consists of eight evolutionarily conserved proteins (SEC3, SEC5, SEC6, SEC8, SEC10, SEC15, EXO70, and EXO84) and is present to tether, dock, and fuse the post-Golgi secretory vesicles at predetermined sites on the plasma membrane, a prerequisite for subsequent SNARE-mediated membrane fusion [2,4,5,6].

In yeast and metazoans, exocyst subunits are encoded by single genes or a few paralogs, but plants exhibit a surprisingly high number of isoforms. This expansion is particularly pronounced for the EXO70 subunit, which in *Arabidopsis* is encoded by 23 distinct loci [7]. Different EXO70 subtypes are considered as specific determinants, endowing unique spatial and functional identities to exocyst complexes through interactions with different vesicle cargos, membrane subdomains, or regulatory proteins [8]. For example, EXO70A1 is crucial for cytokinesis and cell plate formation [9]. Both EXO70B1 and EXO70B2 are involved in plant immune responses [10,11,12,13]; EXO70H4 is involved in trichome cell wall maturation [14]. This functional diversification highlights how the exocyst complex is used to regulate the core processes of plant specific biology.

A member of the EXO70E clade, EXO70E2, was localized to double-membrane structures resembling autophagosomes; these structures were termed EXPOs (exocyst-positive organelles) and were proposed for export from the cell via an exosome-like mechanism [15,16]. EXPOs mediate an unconventional protein secretion (UPS) pathway that allows the release of specific leaderless proteins, independently of the classical endoplasmic reticulum (ER)–Golgi secretory pathway [17,18]. By forming EXPO, EXO70E2 positions itself at a critical cellular intersection, bridging conventional exocytosis, UPS, and potentially stress-responsive extracellular signaling.

Consistently with its role in stress response, EXO70E2 expression is highly responsive to environmental stimuli. Its promoter is active in root tips and hydathodes [8,14], and its transcript level is induced by *Pseudomonas syringae* pv. *lachrymans* (*Pls*) and *Fusarium oxysporum* f. sp. *cucumerinum* Owen (*Foc*) [14]. However, the functional outcome of EXO70E2 activity in plant–pathogen interactions present an interesting paradox. Although its expression is upregulated by immune signals, new evidence suggests it may not function as a straightforward defense promoter. For example, EXO70E2 physically interacts with RIN4 (RPM1-INTERACTING PROTEIN 4), a central regulator and guardee of plant immunity that is targeted by multiple bacterial effectors [19,20]. Our previous work demonstrated that transiently expressed RIN4 (RPM1-INTERACTING PROTEIN 4) recruits EXO70E2 vesicles to the plasma membrane and promotes their transport to the extracellular matrix [21]. We further established that the RING-type E3 ubiquitin ligase BOI (Botrytis Susceptible1 Interactor) targets EXO70E2 for ubiquitin-mediated degradation [22]. This regulation places EXO70E2 within a dynamic negative feedback loop, indicating that its protein levels and activity must be strictly and transiently controlled to maintain cellular homeostasis.

This study aims to address this functional paradox by systematically investigating the consequences of constitutive EXO70E2 overexpression in *Arabidopsis*. We hypothesize that EXO70E2 acts as a critical, dosage-sensitive node that regulates resource allocation between growth and defense. Through detailed phenotypic, cellular, and pathological analyses, we demonstrate that elevated EXO70E2 levels cause severe developmental defects and, contrary to expectations based on its expression pattern, significantly increase susceptibility to the necrotrophic fungus *B. cinerea*. Our work incorporates the novel BOI-mediated regulatory mechanism into a model, in which dysregulated EXO70E2 becomes a susceptibility factor by transferring cellular resources from effective anti-necrotrophic defenses. These findings deepen our understanding of how specific vesicle trafficking components are integrate and disrupt the classical growth–defense trade-offs in plants.

## 2. Materials and Methods

### 2.1. Plant Materials and Growth Conditions

*Arabidopsis* ecotype Columbia-0 (Col-0) was utilized as the wild-type background for all experiments. *Arabidopsis* and *Nicotiana benthamiana* were cultivated in a growth chamber under long-day conditions (16 h light/8 h dark) at 24 °C. *P. syringae* pv *tomato* DC3000 and *B. cinerea* BO5.10, as well as seeds of *Arabidopsis* and *N. benthamiana,* were provided by Professor Zhiyong Gao of the college of life sciences, Wuhan University. We gratefully acknowledge his contribution. For seedling assays, surface-sterilized *Arabidopsis* seeds were plated on half-strength Murashige and Skoog (1/2 MS) medium supplemented with 1% sucrose and solidified with 1.2% agar.

### 2.2. Plasmid Construction and Plant Transformation

The full-length *EXO70E2* coding sequence was amplified and cloned into the binary vector pEarleyGate101, generating a C-terminal YFP-HA fusion under the control of the Cauliflower Mosaic Virus (CaMV) 35S promoter (*35S::EXO70E2-YFP-HA*). For promoter analysis, a 1888 bp fragment upstream of the EXO70E2 start codon was fused to the β-glucuronidase (GUS) reporter gene (*ProEXO70E2::GUS*). Constructs were introduced into the *Agrobacterium tumefaciens* strain GV3101 and transformed into Col-0 via the floral dip method [23]. The *Agrobacterium* strain GV3101 was grown to OD_600_ = 0.8, resuspended in 5% (*w*/*v*) sucrose and 0.05% (*v*/*v*) Silwet L-77, and used to inoculate flower buds for 30 s [24]. During the procedure, T-DNA inserted the target gene into the female gamete genome of the flower buds [25]. For selection of homozygous transgenic lines, T1 generation transgenic plants that exhibit kanamycin or hygromycin were harvested individually to obtain seeds for the T2 generation. From the T2 lines confirmed as single-copy insertion lines via segregation analysis, 18 resistant seedlings were randomly chosen (of which theoretically 1/3 should be homozygous lines) for transplantation and individual harvesting to obtain T3 generation seeds, and homozygous T3 lines were used for subsequent experiments.

### 2.3. Phenotypic Characterization and Root Growth Assays

The gross morphology of four-week-old soil-grown plants was documented. For root growth assays, 10-day-old seedlings grown vertically on 1/2 MS plates were scanned. Primary root lengths were quantified using ImageJ 1 software. To visualize the cellular architecture of the root apical meristem (RAM), root tips were stained with 10 μg/mL of Propidium Iodide (PI) for 5 min and imaged using a confocal laser scanning microscope.

### 2.4. Subcellular Localization and Chemical Treatments

The subcellular distribution of EXO70E2-YFP was observed in the leaf epidermal cells of stable *Arabidopsis* transgenic lines and via transient expression in *N. benthamiana*. Confocal imaging was performed using a Leica SP8 microscope (YFP excitation at 514 nm and detection at 525–575 nm). To assess the response to defense signaling, leaves were treated with the salicylic acid (SA) analog INA for 24 h prior to imaging.

### 2.5. B. cinerea Inoculation and Disease Evaluation

A Neubauer hemocytometer was used to quantify the spore suspension of *B. cinerea*, which was adjusted to 2 × 10^5^ spores/mL. Leaves of 4–5-week-old plants were drop-inoculated with 4 µL of suspension. Disease symptoms and lesion diameters were recorded at 5 days post-inoculation (dpi). For cell death visualization, inoculated leaves were stained with trypan blue at 0, 24, and 48 hpi. Fungal biomass was quantified via quantitative PCR (qPCR) using primers specific to the *B. cinerea* cutinase A (*Bc*CutA) gene, normalized to the *Arabidopsis* α-shaggy kinase (*At*ASK) gene.

### 2.6. Histochemical GUS Staining and Stress Assays

GUS activity in *ProEXO70E2::GUS* lines was detected by incubating tissues in a staining buffer [100 mM sodium phosphate (pH 7.0), 10 mM EDTA, 0.5 mM K_3_Fe(CN)_6_, 0.5 mM K_4_Fe(CN)_6_, and 1 mM X-Gluc] at 37 °C for 12 h. Tissues were cleared with 75% ethanol before imaging. Stress Assays were performed as previously described [26].

### 2.7. Protein Analysis and Quantitative PCR (qPCR)

Total protein was extracted in a buffer containing 50 mM Tris-HCl (pH 7.5), 150 mM NaCl, 1 mM EDTA, and 1% Triton X-100. Immunoblotting was performed using anti-HA (Roche, Basel, Switzerland, #11867423001)(1:5000) and anti-β-actin (Abbkine, Wuhan, China, #A01050-2)(1:3000) antibodies. Proteins were separated on an 8% SDS-PAGE gel to ensure clear resolution between the 85 kDa EXO70E2-YFP-HA protein and the 43 kDa β-actin internal control. Fungal biomass was quantified by genomic DNA-based qPCR. The *B. cinerea Bc*CutA gene levels were normalized to the *Arabidopsis At*ASK gene. Relative expression or biomass was calculated using the 2^−ΔΔCt^ method.

### 2.8. Statistical Analysis

All experiments were performed with at least three biological replicates. Data were analyzed using SPSS 16.0. Quantitative differences were evaluated using one-way ANOVA followed by Duncan’s multiple-range test, or two-tailed Student’s t-tests. *p*-values < 0.05 were considered statistically significant.

## 3. Results

### 3.1. Constitutive Overexpression of EXO70E2 in Arabidopsis Results in Severe Developmental Abnormalities

**Figure 1:**
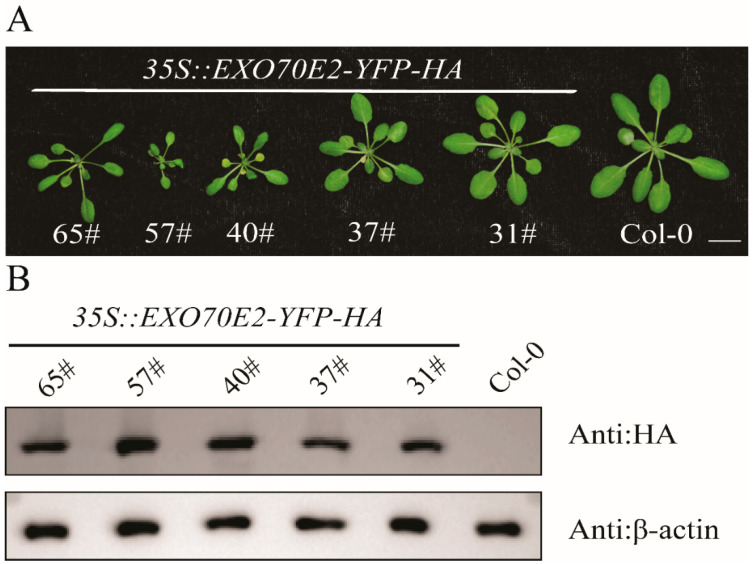
Overexpression of EXO70E2 in *Arabidopsis* leads to severe developmental abnormalities. (**A**) The phenotypes of four-week-old *35S::EXO70E2-YFP-HA* transgenic plants in the wild-type (Col-0) background. Col-0 plants were employed as controls. # represents *Arabidopsis* homozygous line. (**B**) The protein levels of EXO70E2 were detected with the anti-HA antibody. β-actin was used as the loading control. Bar = 1000 μm.

To investigate the function of EXO70E2 in plants, we constructed stable transgenic *Arabidopsis* lines expressing the EXO70E2-YFP-HA fusion gene driven by the constitutive cauliflower mosaic virus (CaMV) 35S promoter. Five independent, homozygous T3 lines (31#, 37#, 40#, 57#, 65#) were isolated and propagated. All overexpression lines (hereafter EXO70E2-OE) exhibited consistent and severe phenotypic deviation with wild-type (Col-0) plants (Figure 1A). The most prominent features were pronounced etiolation, indicating a decrease in chlorophyll content, and obvious dwarfism, suggesting fundamental damage to cell expansion or division. Immunoblot analysis confirmed the high-level accumulation of EXO70E2 protein in transgenic lines (Figure 1B). The correlation between the presence of the transgene product and the observed developmental defects suggests that the etiolated and dwarfed phenotypes directly caused by elevated EXO70E2 protein levels, rather than random insertion effects.

### 3.2. EXO70E2 Localizes to Punctate Cytoplasmic Structures, and Its Fluorescence Intensity Increases Under Salicylic Acid Signaling

**Figure 2:**
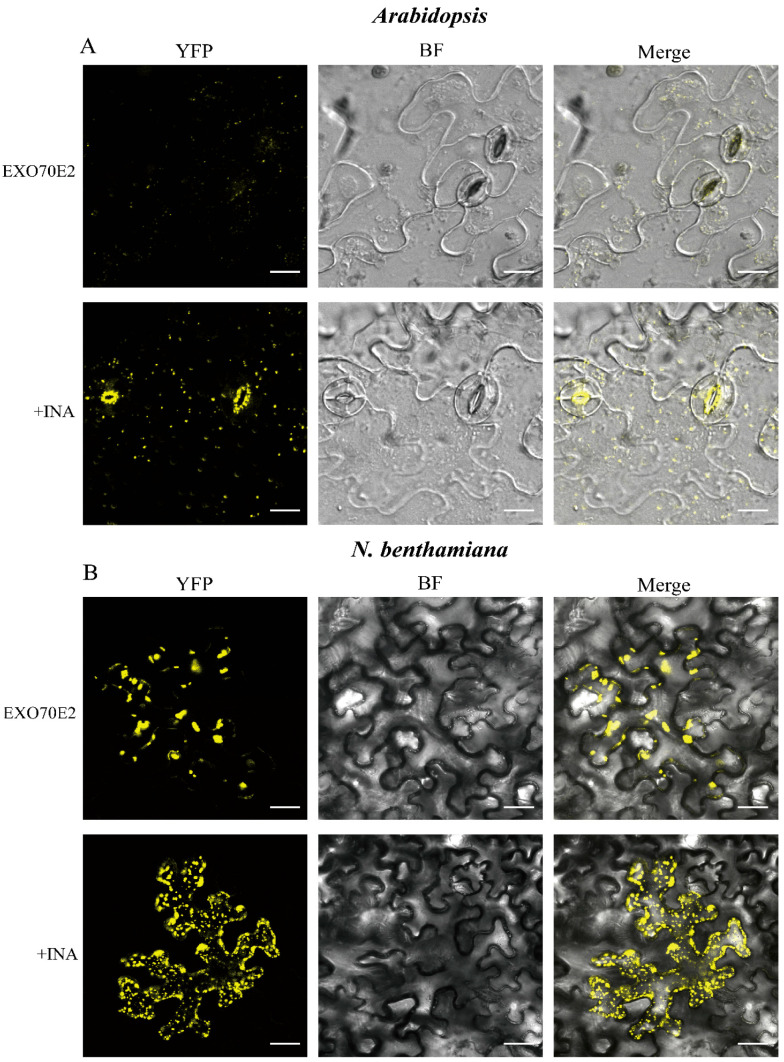
EXO70E2 localizes to punctate cytoplasmic structures, and its fluorescence intensity increases in response to salicylic acid signals. INA treatment of transgenic *Arabidopsis* (**A**) and *N. benthamiana*. (**B**) Leaves can significantly enhance the fluorescence intensity of EXO70E2-YFP-HA. Bars = 25 μm.

A protein’s function is closely linked to its subcellular localization. Therefore, we examined the subcellular localization of EXO70E2 in *Arabidopsis* and *N. benthamiana*. Confocal microscopy of leaf epidermal cells from the representative high-expression line (57#) revealed that the EXO70E2-YFP signal was concentrated in numerous distinct punctate structures (Figure 2A, upper panel). Similar results were obtained in the leaves of *N. benthamiana* (Figure 2B, upper panel). The observation corroborates previous findings that EXO70E2 is a core organizer of the exocyst-positive organelles (EXPOs) [15]. Treatment with benzothiadiazole (BTH), a functional analog of salicylic acid, strongly induces autophagy in *Arabidopsis*. Following BTH or SA signal induction, EXO70E2 colocalizes with the autophagy marker ATG8 [27]. This finding indicates a correlation between salicylic acid signaling and EXO70E2. Furthermore, we treated INA, a functional analog of SA [28], in transgenic *Arabidopsis* and *N. benthamiana* leaves. We observed a significant and reproducible increase in YFP fluorescence intensity relative to the control (Figure 2A, lower panel, and Figure 2B, lower panel), indicating that the stability and accumulation of EXO70E2 protein may be post-transcriptionally regulated by SA-mediated defense signaling pathways. In summary, these data establish a critical link between the protein’s localization/abundance and plant immune response.

### 3.3. Spatiotemporal and Stress-Responsive Regulation of the EXO70E2 Promoter

**Figure 3:**
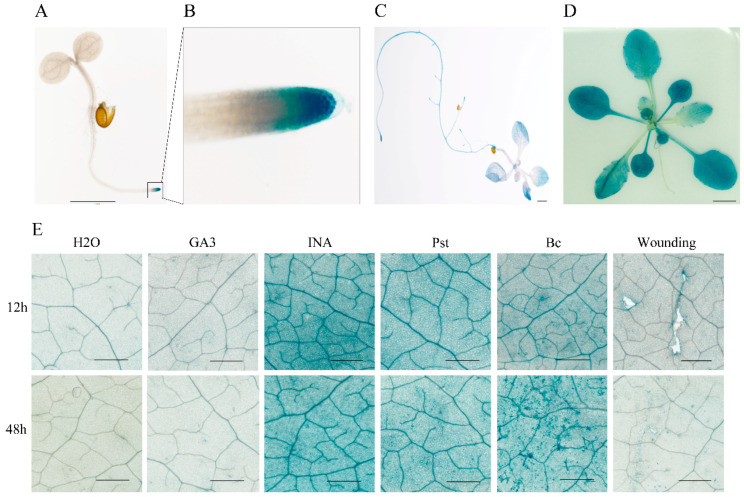
Tissue-specific expression of *EXO70E2*, and its biotic and abiotic-induced expression. (**A**) Expression pattern of *EXO70E2* in *ProEXO70E2::GUS* transgenic *Arabidopsis*. Tissues of *ProEXO70E2::GUS* transgenic plants including 3-day-old seedlings (**A**), the root tip of the main root (**B**), 7-day-old seedlings (**C**), and the rosette leaf of a 3-week-old plant (**D**). Bars = 1000 μm. (**E**) GUS staining of *ProEXO70E2::GUS* plant leaves after multiple stress treatments. Four-week-old plants were treated with water (H_2_O), gibberellin (GA3), INA, *P. syringae* pv. *tomato* (*Pst*), *B. cinerea* (*B_C_*) or wounding, for 12 h and 48 h, respectively. Bars = 1000 μm.

To dissect temporal and spatial expression patterns, transgenic plants harboring a 1888 bp promoter-GUS reporter construct were generated, and the promoter activity was assayed by histochemical GUS staining. Histochemical GUS staining revealed a dynamic, developmentally regulated expression pattern (Figure 3A–D). In 3-day-old seedlings, GUS activity was most prominent in the root tip (Figure 3A,B). By 7 days, this pattern had expanded to include the apical tips of main leaf veins and the hypocotyl, while strong staining persisted in the root tips (Figure 3C). In mature rosette leaves of 3-week-old plants, expression was evident throughout the leaf blade and petioles (Figure 3D). This spatial progression implies a role for EXO70E2 in both meristematic activity and mature tissue function.

To further examine the promoter activities of the EXO70E2 in response to signaling molecules and environmental stress, 4-week-old *ProEXO70E2::GUS* plants were subjected to various biotic and abiotic stimuli. Leaves were treated with water (control), gibberellin (GA3), or INA or infected with *P. syringae* pv. *tomato* (*Pst*) or *B.* cinerea (*Bc*), or they were mechanically wounded. GUS activity was monitored at 12 and 48 h post-treatment (hpt) (Figure 3E). A clear induction of GUS staining occurred specifically in response to INA treatment and to infection by both *Pst* and *B. cinerea* at both time points. In contrast, GA3 treatment or wounding elicited only a minimal or inconsistent response. These results indicate that EXO70E2 is a pathogen-responsive gene whose transcription is activated by defense signals associated with the salicylic acid pathway. In addition, the induction of *B. cinerea* is particularly interesting as it indicates the potential role of EXO70E2 in a broader spectrum of biological interactions.

### 3.4. EXO70E2 Overexpression Dose-Dependently Suppresses Primary Root Elongation

**Figure 4:**
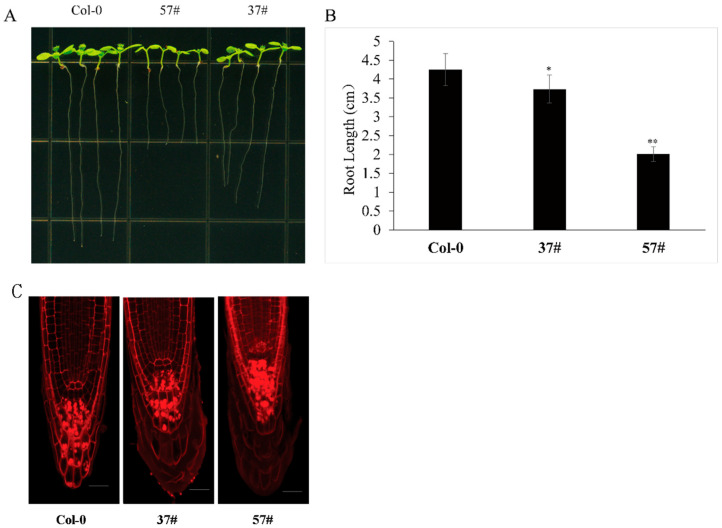
EXO70E2 suppresses primary root elongation in a dose-dependent manner. (**A**) Overexpression of EXO70E2 in the transgenic lines 37# and 57# resulted in shorter primary roots than those of the wild-type (Col-0) at 10 days post-germination. (**B**) Quantitative analysis of primary root length. Data represent means ± SD (n ≥ 24). Significant differences were determined by one-way ANOVA followed by Duncan’s multiple-range test (* *p* < 0.05; ** *p* < 0.01). (**C**) Confocal laser scanning microscopy images of root tips stained with Propidium Iodide (PI). Note the altered cellular architecture and meristematic zone organization in the OE lines (37# and 57#). Bars = 20 μm.

The high expression of *EXO70E2* in root tips (Figure 3B) allowed us to explore its role in root development. Primary root length was measured in 10-day-old seedlings of Col-0 and two EXO70E2-OE lines (37# and 57#). Root growth was significantly inhibited in both overexpression lines (Figure 4A). The more severe phenotype occurred in line 57#, which accumulated higher EXO70E2 protein levels (Figure 1B), with a mean root length of 2.01 cm—a 53% reduction relative to the 4.25 cm observed in Col-0. Line 37#, a moderate expresser, displayed a significant but less pronounced inhibition, reaching 3.73 cm (Figure 4B). The magnitude of root growth inhibition correlated positively with EXO70E2 protein accumulation levels, demonstrating a dose-dependent inhibitory effect on primary root elongation. In a word, these results suggest that EXO70E2-mediated processes play a specific and sensitive role in regulating root cell expansion or meristem activity. To further investigate the cellular basis of this growth defect, we performed Propidium Iodide (PI) staining to visualize the root apical meristem (RAM) architecture [29]. PI staining highlights the cell walls and differentiates the developmental zones of the root tip. In Col-0, the root tip maintained a highly organized cellular arrangement with a clearly defined quiescent center and distinct layers of columella cells. In contrast, the shorter-rooted lines (particularly 57#) showed altered cellular patterning at the apex (Figure 4C). The intensified fluorescence and tighter cell packing in the meristematic zone suggest that the observed reduction in root length was likely driven by a decrease in the rate of cell division or a premature transition from cell proliferation to differentiation within the RAM.

### 3.5. Elevated EXO70E2 Levels Increase Susceptibility to B. cinerea, Promoting Fungal Colonization and Host Cell Death

**Figure 5:**
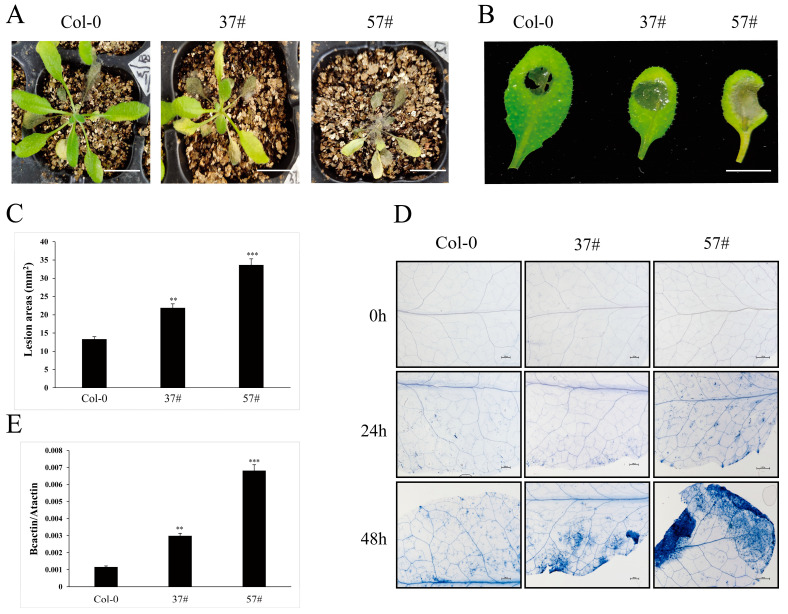
The *Arabidopsis* EXO70E2-OE lines exhibited increased susceptibility to *B. cinerea*. (**A**) Plants at approximately four weeks of age were sprayed with a *B. cinerea* spore suspension (2 × 10^5^ spores/mL), and a representative image was captured at 5 days post-inoculation (dpi). Bar = 1000 μm. (**B**) Detached leaves from each line were inoculated with a 4 µL droplet of the same spore suspension, and disease progression was assessed after 2 days. Bar = 1000 μm. (**C**) Lesion areas were measured at 5 dpi, with at least 20 lesions measured per line. Differences in lesion size were evaluated by Tukey’s HSD test (** *p*  <  0.01; *** *p*  <  0.001). The experiment was performed in triplicate with consistent results. (**D**) Trypan blue staining of leaves at different time points after spray inoculation with *B. cinerea*. Bar = 1000 μm. (**E**) Fungal biomass in planta was assessed by qPCR using *B. cinerea*- and *Arabidopsis*-specific primers. The *B. cinerea* gene *Bc*CutA was quantified from extracted genomic DNA, with the *Arabidopsis* α-shaggy kinase gene (*At*ASK) serving as an internal reference. Data represent means ± SD of three biological replicates. Statistical significance was determined by two-tailed Student’s t-tests, comparing *Bc*CutA levels between Col-0 and EXO70E2-OE plants at each time point (** *p*  <  0.01; *** *p*  <  0.001).

Due to the transcriptional induction of *EXO70E2* by *B. cinerea* (Figure 3E), we investigated its functional role in this host–pathogen interaction. Plants or detached leaves from 4–5-week-old Col-0 and EXO70E2-OE (37# and 57#) were sprayed or drop-inoculated with a conidial suspension of *B. cinerea*, respectively. By 5 days post-inoculation (dpi), EXO70E2-OE lines (37# and 57#) exhibited exacerbated necrotic symptoms and significantly larger lesion areas compared to the wild-type Col-0 (Figure 5A–C).

We further carried out trypan blue staining at 0, 24, and 48 h post-inoculation (hpi) to observe leaf cell death. The trypan blue staining results of infected leaves showed that the number of dead cells in the transgenic line increased faster than those in Col-0, and larger and expanding lesion areas were observed beyond the inoculation sites at various time points (Figure 5D), showing that the elevated EXO70E2 not only permitted larger lesion formation but also accelerated and expanded host tissue necrosis caused by *B. cinerea*.

To obtain direct molecular evidence of altered fungal proliferation, we quantified fungal biomass *in planta* using quantitative PCR (qPCR). We measured the relative abundance of the *B. cinerea*-specific cutinase A (*Bc*CutA) gene, normalized to the *Arabidopsis* α-shaggy kinase (*At*ASK) gene, at 48 hpi. The qPCR analysis showed that the expression level of CutA is significantly higher in two OE lines (37# and 57#) compared to Col-0 (Figure 5D). In summary, the increased lesion size, accelerated host cell death, and elevated fungal biomass collectively demonstrate that constitutive EXO70E2 overexpression compromises *Arabidopsis* innate immunity, thereby enhancing its susceptibility to *B. cinerea*.

## 4. Discussion

Our experiments show that constitutive overexpression of EXO70E2 in *Arabidopsis* produces a dose-dependent phenotype characterized by etiolation, dwarfism, and suppressed primary root elongation (Figure 1 and Figure 4), demonstrating a non-redundant, growth-suppressive function role for EXO70E2. This phenotype is consistent with the established function of polarized exocytosis directed by specific EXO70 subtypes, in driving cell expansion and organogenesis [3,7]. We propose that the observed growth defects arise not from generalized toxicity but from a specific disruption of secretory homeostasis. This effect contrasts with the loss-of-function phenotype of other EXO70 genes, such as exo70a1, which also exhibits root defects [30]. This comparison underscores the critical importance of precise stoichiometry within the exocyst complex for its developmental function.

EXO70E2-YFP displayed a punctate localization pattern (Figure 2), consistent with its established association with EXPOs and other endomembrane compartments [15]. Following INA treatment, its signal intensity increased (Figure 2), indicating a post-transcriptional regulatory link between EXO70E2 and SA signaling. As a key defense hormone against biotrophic pathogens [31], SA could stabilize the EXO70E2 protein or reduce its turnover. This finding complements our recent work demonstrating that RIN4 can promote the transport of the EXO70E2 to the extracellular matrix [21]. Consequently, EXO70E2 acts as a direct link between SA signaling and a dedicated secretory pathway.

The dose-dependent inhibition of primary root elongation in *EXO70E2-OE* lines (Figure 4A,B) highlights the critical role of this exocyst subunit in modulating plant architecture. The significant reduction in root length—exceeding 50% in the high-expression line 57#—suggests that EXO70E2 serves as a potent negative regulator of root development when expressed constitutively. To elucidate the cellular basis of this stunted growth, we analyzed the root apical meristem (RAM) using Propidium Iodide (PI) staining. The cellular patterning observed in Figure 4C reveals that the shortened-root phenotype is likely driven by fundamental disruptions within the meristematic zone. Considering root growth is determined by a precise equilibrium between cell division in the meristem and cell expansion in the elongation zone. The altered cellular architecture and intensified cell packing in the *EXO70E2-OE* root tips suggest that excessive EXO70E2 protein may trigger a premature transition from proliferation to differentiation or impair the rate of mitotic activity. As a core organizer of EXPOs, overabundant EXO70E2 might cause a massive misdirection of secretory vesicles, diverting essential cell-wall biosynthetic enzymes and plasma membrane components away from the rapidly expanding cell surfaces required for elongation.

Moreover, EXO70E2-OE plants exhibit increased susceptibility to *B. cinerea* (Figure 5). Our data indicate that EXO70E2 does not directly execute SA-mediated resistance. Instead, EXO70E2 appears to function as a susceptibility factor or a negative regulator of the jasmonate/ethylene (JA/ET)-mediated defense pathway, which is effective against necrotrophic pathogens [32,33,34]. This paradox may be explained by the growth-defense trade-off and the post-translational regulation of EXO70E2. A hyperactive EXPO pathway, driven by EXO70E2 overexpression, acts as a substantial metabolic sink for energy, lipids, and proteins. This constitutive drain leaves the plant unable to mobilize the resource-intensive defenses required to combat *B. cinerea*. Susceptibility thus arises not from direct suppression of defense genes by EXO70E2, but from the indirect starvation of the defense program. Support for this model comes from the E3 ubiquitin ligase BOI, which targets EXO70E2 for degradation [22] and is induced by *B. cinerea* and stress [35]. This interaction establishes a critical negative feedback loop. In wild-type plants, transient pathogen-triggered pulses of EXO70E2 likely enable rapid responses, yet subsequent BOI-mediated degradation ensures that the response is self-limiting and prevents sustained resource transfer. The overexpressed plants in this study appear to bypass this regulatory checkpoint. Their constitutively high EXO70E2 levels escape BOI-mediated control, resulting in a permanent state of resource misallocation that cripples effective defense against necrotrophic fungus *B. cinerea*.

Furthermore, positioning EXO70E2 within the broader EXO70 family reveals its distinct role. Unlike EXO70B2, a positive regulator of PTI that is degraded by the E3 ligase PUB22 to attenuate immunity [36,37], EXO70E2 functions as a negative regulator of defense against necrotrophic and is targeted for degradation by BOI [22]. This contrast illustrates the precise specificity and opposing functions that different EXO70 isoforms can adopt within immune regulation. Although both are regulated by ubiquitination, their consequences for plant defense differ, reflecting the complexity of vesicle trafficking in tailoring immune responses to specific pathogen lifestyles.

## 5. Conclusions

In this study, we found that EXO70E2 overexpression leads to severe etiolation and dwarfism, accompanied by dose-dependent inhibition of primary root elongation. The YFP-labeled EXO70E2 localizes to distinct punctate structures. Histochemical analysis shows EXO70E2 expression in root tips and leaf vasculature, and its promoter activity is strongly induced by the salicylic acid analog INA and pathogen infection. Further function dissection indicates that EXO70E2-overexpressing plants exhibit enhanced susceptibility to the necrotrophic fungus *B. cinerea*.

## Data Availability

The original contributions presented in this study are included in the article. Further inquiries can be directed to the corresponding author.
